# No Association of Obesity and Type 2 Diabetes Mellitus Related Genetic Variants With Colon Cancer

**DOI:** 10.4021/gr2009.11.1323

**Published:** 2009-11-20

**Authors:** Cheryl L. Thompson, Sarah J. Plummer, Thomas C. Tucker, Graham Casey, Li Li

**Affiliations:** a Departments of Family Medicine, Case Western Reserve University/University Hospitals of Cleveland, 10900 Euclid Ave, Cleveland, OH 44106, USA; b Epidemiology and Biostatistics, Case Western Reserve University/University Hospitals of Cleveland, 10900 Euclid Ave, Cleveland, OH 44106, USA; c The Case Center for Transdisciplinary Research on Energetics and Cancer, Case Comprehensive Cancer Center, 10900 Euclid Ave, Cleveland, OH 44106, USA; d Department of Preventive Medicine, University of Southern California, 1975 Zonal Ave, Los Angeles, CA 90089, USA; e Cancer Control Program, University of Kentucky, 2365 Harrodsburg Rd, Lexington, KY 40504, USA

**Keywords:** Obesity, Type 2 diabetes, Colon cancer, Single nucleotide polymorphisms, Association

## Abstract

**Background:**

Obesity and type 2 diabetes mellitus (T2D) are known risk factors for colon cancer. Recent reports from a number of genome wide association studies (GWAS) have identified several single nucleotide polymorphisms (SNPs) associated with obesity and T2D. Here we tested the hypothesis that these SNPs may also be associated with risk of colon cancer.

**Methods:**

We genotyped nine SNPs reported in GWAS of obesity and/or T2D, including SNPs in HHEX, KCNJ11, SLC30A8, FTO, CDKN2, CDKAL1, TCF2, and the rs9300039 SNP in an intergenic region, in 561 colon cancer cases and 721 population controls.

**Results:**

None of these SNPs were statistically significantly associated with colon cancer in our sample.

**Conclusions:**

Overall, these results suggest that these obesity and T2D genetic susceptibility loci are unlikely to influence the risk of colon cancer.

## Introduction

Obesity is now considered as a ‘probable’ cause for colon cancer [1], and insulin resistance with resulting metabolic perturbation is believed to be the key mechanism underlying the obesity-colon cancer link [2, 3]. Colon cancer and type 2 diabetes mellitus (T2D) share a remarkably similar risk factor profile, including obesity, high energy intake, a Western diet and physical inactivity. In addition, T2D itself is directly associated with colon cancer [2]. It is thus plausible that genetic susceptibility loci for obesity and T2D may also predispose to the development of colon cancer.

Genome-wide association studies (GWAS) have recently identified a number of novel single nucleotide polymorphisms (SNPs) that confer susceptibility to T2D [3-7] or obesity [8-11]. Although the functionality of these genetic variants is largely unknown, many of the genes in which the SNPs are located are known to be oncogenic or involved in molecular pathways implicated in carcinogenesis. For example, *HHEX* encodes a transcription factor in the homeobox family, and there is evidence that this gene can act as an oncogene [12]. *CDKN2* is a well known tumor suppressor gene [13] which stabilizes the tumor suppressor p53. Mutations of the *CDKN2* gene have been reported in numerous tumor types [14]. *TCF2* (*HNF1B*), like *HHEX*, encodes a homeobox transcription factor. *TCF2* has been recognized as a key player in carcinogenesis, especially in prostate cancer [15, 16].

To date, whether these genetic variants may also be associated with risk of cancer has not been investigated. We therefore tested this hypothesis in a population-based case-control study of colon cancer.

## Materials and Methods

### Study population

The study population is described elsewhere [17]. In brief, we recruited 561 incident colon cancer cases and 721 population controls. Colon cancer cases were identified via the Kentucky Surveillance, Epidemiology and End Results (SEER) Cancer Registry within 6 months of diagnosis. Controls were recruited via random digit dialing. All individuals were asked to donate a sample of blood for genomic DNA. We used the National Cancer Institute Colon Risk Factor Questionnaire (http://epi.grants.cancer.gov/documents/CFR/center_questionnaires/Colon/LA/ColonRiskFactor_USC.pdf) to collect complete lifestyle and behavioral risk factors, including demographic variables, family history of colorectal cancer and non-steroidal anti-inflammatory drug (NSAID) use. Body mass index was calculated based on self-reported height and weight. The response rate was approximately 72% cases, and 63% for eligible controls. The study was approved by the institutional review boards of Case Western Reserve University/University Hospitals Case Medical Center, the University of Kentucky, Lexington, and the University of Southern California, Los Angeles.

### Selection of SNPs and genotyping

We selected nine SNPs previously identified to be associated with obesity (rs9939609 and rs8050136 in FTO) [8, 11] or T2D (rs1111875 in HHEX, rs5219 in KCNJ11, rs9300039, rs13266634 in SLC30A8, rs10811661 in CDKN2, rs7754840 in CDKAL1 and rs4430796 in TCF2) [3-7], as reported in GWAS through September 2007. All SNPs were genotyped by the TAQMAN assay using predesigned probe/primer sets from Applied Biosystems. Two percent of samples were repeated giving 100% concordance.

### Statistical analyses

We first tested the fit of each SNP to Hardy-Weinberg proportions using a Chi-square goodness of fit. Since cases were specifically ascertained to a disease we hypothesize would be associated with these SNPs, we only used controls in this analysis.

To examine the association of each SNP with colon cancer, we initially assessed the univariate (individual SNP) association via a chi-square test. We further obtained odds ratios (OR) via a logistic regression accounting for age, sex and race (base model) as well as after fully adjusting for age, sex, race, BMI, non-steroidal anti-inflammatory drug (NSAID) use, and family history of colorectal cancer (full model). In the logistic regressions, we tested three genetic models (dominant, recessive or additive). For the additive model, each individual was coded as 0, 1 or 2, representing the number of risk (less frequent) alleles they possessed for each SNP. Dominant models were coded as 1 if the individual possessed 1 or 2 copies of the risk allele, 0 otherwise, whereas the recessive models were coded as 1 only if the individual possessed two copies of the risk allele, and 0 otherwise. To address possible spurious associations resulting from population stratification, we also performed analyses restricted to Caucasians only. SAS Version 9.1 (SAS Institute, Inc., Cary, North Carolina) was used to calculate all statistics. An α = 0.05 was used to declare statistical significance.

## Results

On average, cases were older and more likely to be male than controls (Table 1). Compared to controls, cases had a higher mean BMI (p = 0.0077), were more likely to report a family history of colorectal cancer (24.0% vs. 15.4%, p = 0.0014), more likely to be diagnosed with diabetes (20.9% vs. 15.6%, p = 0.019), and less likely to regularly use NSAIDs (p = 0.16).

**Table 1:** Descriptive Characteristics of Kentucky Colon Cancer Study Population

	Cases (N = 561)	Controls (N = 721)	p*
Gender – N (%)			< 0.001
Male	266 (47.3)	274 (38.0)	
Female	295 (52.7)	447 (61.0)	
Mean (stddev) age	62.8 (10.7)	58.3 (10.9)	< 0.001
Race – N (%)			0.24
African-American	27 (4.8)	23 (3.2)	
Caucasian	526 (93.8)	678 (94.0)	
Other	8 (1.4)	15 (2.1)	
Unknown	0 (0)	5 (0.7)	
Body Mass Index (kg/m^2^)	29.3 (6.1)	28.3 (6.2)	0.0077
Diabetes Diagnosis (%)**			0.019
Yes	105 (18.7)	105 (14.6)	
No	397 (70.8)	568 (78.8)	
Unknown	59 (10.5)	48 (6.7)	
Family History‡			0.0014
Yes	94 (16.8)	72 (10.0)	
No	297 (52.9)	395 (54.8)	
Unknown	170 (30.3)	254 (35.2)	
Regular NSAID Use^†^			0.16
Yes	235 (41.9)	306 (42.4)	
No	131 (23.4)	138 (19.1)	
Unknown	195 (38.8)	277 (38.4)	
HHEX – rs1111875			0.13
CC	217 (38.7)	241 (33.4)	
CT	255 (45.5)	348 (48.3)	
TT	88 (15.7)	131 (18.2)	
No call	1 (0.2)	1 (0.1)	
KCNJ11-rs5219			0.39
CC	226 (40.3)	304 (42.2)	
CT	265 (47.2)	316 (43.8)	
TT	68 (12.1)	100 (13.9)	
No call	2 (0.4)	1 (0.1)	
rs9300039			0.73
AA	9 (1.6)	8 (1.1)	
AC	103 (18.4)	136 (18.9)	
CC	448 (79.9)	576 (79.9)	
No call	1 (0.2)	1 (0.1)	
SLC30A8 – rs1326634			0.48
CC	274 (48.9)	329 (45.6)	
CT	230 (41.0)	319 (44.2)	
TT	55 (9.8)	71 (9.9)	
No call	2 (0.4)	2 (0.3)	
FTO-rs9939609 AA			0.66
AA	90 (16.0)	121 (16.8)	
AT	252 (44.9)	336 (46.6)	
TT	217 (38.7)	261 (36.2)	
No call	2 (0.4)	3 (0.4)	
FTO-rs8050136			0.74
AA	91 (16.2)	119 (16.5)	
AC	250 (44.6)	336 (46.6)	
CC	217 (38.7)	265 (36.8)	
No call	3 (0.5)	1 (0.1)	
CDKN2 – rs10811661			0.34
CC	21 (3.7)	19 (2.6)	
CT	142 (25.3)	202 (28.0)	
TT	395 (70.4)	500 (69.4)	
No call	3 (0.5)	0 (0)	
CDKAL1 – rs7754840			0.75
GG	241 (43.0)	325 (45.1)	
CG	254 (45.3)	313 (43.4)	
CC	64 (11.4)	81 (11.2)	
No call	2 (0.4)	2 (0.3)	
TCF2 – rs4430796			0.81
AA	141 (25.1)	178 (24.7)	
AG	297 (52.9)	374 (51.9)	
GG	122 (21.7)	168 (23.3)	
No call	1 (0.2)	1 (0.1)	

*Univariate (t-test or chi-square) p-value for association with colon cancer. For family history, type 2 diabetes diagnosis and NSAID use, this is a test of difference in positive vs. negative responses between cases and controls, ignoring unknown or missing data. **Self-reported physician diagnosis of type 2 diabetes ^†^Regular non-steroidal anti-inflammatory drug (NSAID) use was defined as reporting ever use of NSAIDs at least twice a week for at least six months. ‡Family history of colorectal cancer.

All SNPs conformed to Hardy-Weinberg proportions among the controls (p > 0.05). We found no statistically significant association for any of the SNPs in univariate analyses (Table 1). After adjustment for potential confounders, all associations remained non-significant (Table 2). When the analyses were restricted to Caucasians, we found very similar results. For brevity, we have only presented results from the entire sample here.

**Table 2:** Descriptive Characteristics of Kentucky Colon Cancer Study Population

Gene	SNP	Base Model*		Full Model†	
	Case/Control	OR	p‡	OR	p‡
**HHEX**	**rs1111875**				
CC	217/241	1.0 (ref)	0.20	1.0 (ref)	0.22
CT	255/348	0.80 (0.62 - 1.03)		0.79 (0.60 - 1.03)	
TT	88/131	0.85 (0.60 - 1.19)		0.87 (0.61- 1.24)	
TT vs. CC/CT		0.97 (0.52 - 3.10)	0.84	1.00 (0.72 - 1.37)	0.98
CT/TT vs. CC		0.81 (0.64 - 1.03)	0.089	0.81 (0.63 - 1.04)	0.097
**KCNJ11**	**rs5219**				
CC	226/304	1.0 (ref)	0.93	1.0 (ref)	0.32
CT	265/316	1.15 (0.90 - 1.47)		1.20 (0.92 - 1.56)	
TT	68/100	0.92 (0.63 - 1.32)		0.98 (0.67 - 1.56)	
TT vs. CC/CT		0.85 (0.60 - 1.20)	0.35	0.89 (0.62 - 1.27)	0.89
CT/TT vs. CC		1.10 (0.87 - 1.39)	0.44	1.15 (0.90 - 1.47)	0.28
—	**rs9300039**				
CC	448/576	1.0 (ref)	0.64	1.0 (ref)	0.85
AC	103/136	1.04 (0.78 - 1.41)		1.03 (0.75 - 1.41)	
AA	9/8	1.28 (0.48 - 3.45)		1.35 (0.46 - 3.95)	
AA vs. CC/CA		1.28 (0.47 - 3.47)	0.63	1.35 (0.46 - 3.92)	0.59
AC/AA vs. CC		1.06 (0.79 - 1.41)	0.71	1.05 (0.77 - 1.42)	0.77
**SLC30A8**	**rs13266634**				
CC	274/329	1.0 (ref)	0.60	1.0 (ref)	0.61
CT	230/319	0.92 (0.72 - 1.18)		0.88 (0.68 - 1.14)	
TT	55/71	0.94 (0.63 - 1.41)		0.99 (0.65 - 1.51)	
TT vs. CC/CT		0.98 (0.67 - 1.44)	0.94	1.05 (0.70 - 1.57)	0.81
CT/TT vs. CC		0.93 (0.74 - 1.17)	0.52	0.90 (0.71 - 1.15)	0.40
**FTO**	**rs9939609**				
TT	217/261	1.0 (ref)	0.35	1.0 (ref)	0.40
AT	252/336	0.90 (0.69 - 1.15)		0.84 (0.65 - 1.10)	
AA	90/121	0.86 (0.62 - 1.22)		0.84 (0.58 - 1.19)	
AA vs. TT/AT vs. AA		0.88 (0.70 - 1.12)	0.54	0.92 (0.66 - 1.27)	0.61
AT/AA vs. TT		0.92 (0.67 - 1.25)	0.32	0.84 (0.65 - 1.08)	0.17
**FTO**	**rs8050136**				
CC	217/261	1.0 (ref)	0.42	1.0 (ref)	0.48
AC	250/336	0.90 (0.70 - 1.16)		0.85 (0.65 - 1.11)	
AA	91/119	0.89 (0.64 - 1.25)		0.87 (0.61 - 1.11)	
AA vs. CC/AC		0.90 (0.71 - 1.14)	0.72	0.86 (0.67 - 1.83)	0.23
AC/AA vs. CC		0.95 (0.70 - 1.29)	0.37	0.95 (0.69 - 1.32)	0.76
**CDKN2**	**rs10811661**				
TT	395/500	1.0 (ref)	0.93	1.0 (ref)	0.086
CT	142/202	0.88 (0.68 - 1.15)		0.85 (0.65 - 1.12)	
CC	21/19	1.59 (0.83 - 3.03)		1.88 (0.93 - 3.81)	
CC vs. TT/CT		1.65 (0.86 - 3.16)	0.13	1.97 (0.98 - 3.97)	0.057
CT/CC vs. TT		0.94 (0.73 - 1.21)	0.63	0.93 (0.71-1.21)	0.56
**CDKAL1**	**rs7754840**				
GG	241/325	1.0 (ref)	0.35	1.0 (ref)	0.34
CG	254/313	1.15 (0.90 - 1.47)		1.21 (0.94 - 1.57)	
CC	64/81	1.11 (0.76 - 1.61)		1.16 (0.78 - 1.73)	
CC vs. GG/CG		1.04 (0.73 - 1.50)	0.87	1.05 (0.72 - 1.53)	0.80
CG/CC vs. GG		1.14 (0.91 - 1.44)	0.25	1.20 (0.94 - 1.53)	0.15
**TCF2**	**rs4430796**				
AA	141/178	1.0 (ref)	0.39	1.0 (ref)	0.74
AG	297/374	0.94 (0.71 - 1.25)		0.95 (0.71 - 1.28)	
GG	122/168	0.86 (0.62 - 1.20)		0.87 (0.61 - 1.24)	
GG vs. AA/AG		0.90 (0.68 - 1.18)	0.43	0.90 (0.67 - 1.20)	0.48
AG/GG vs. AA		0.92 (0.70 - 1.20)	0.53	0.92 (0.70 - 1.23)	0.60

*Logistic regression controlling for age, sex and race (558 cases and 698 controls). †Logistic regression controlling for age, sex, race, family history of colorectal cancer, non-steroidal anti-inflammatory. drug (NSAID) use and body mass index (BMI) (503 cases and 670 controls with complete data). ‡p-value of additive, dominant or recessive genetic model.

## Discussion

In this study, we found no association with colon cancer risk of a number of SNPs identified through T2D or obesity GWAS. Although many of the SNPs discovered through GWAS have been noted to have small effect sizes, we have at least 80% power to detect an odds ratio as low as 1.26, which is typical of most variants identified through GWAS, using the average minor allele frequency observed in these SNPs (33%) and assuming an additive model. Most of the SNPs included here are common variants with a relatively high frequency (> 10%) of the minor allele homozygotes. Thus it is unlikely that lack of statistical power could have obscured true association for these genetic variants. However, caution needs to be exercised for the KCNJ11 rs930039 and CDNK2 rs10811661 SNPs, for which the frequencies of the minor allele homozygotes are fairly low, limiting our power to detect an effect of these SNPs that truly follows a recessive mode of inheritance. Furthermore, we were able to replicate the association of the *CDKAL1* rs7754840 SNP with self-reported T2D in this population, which compared to the most common GG genotype, individuals in our population with either the CG or CC genotype showed evidence of an increased risk of T2D (OR_CG_ = 1.53, 95% CI = 1.08-2.16; OR_CC_ = 1.63, 95% CI = 0.97-2.73; p_dom_ = 0.0095 and p_add_ = 0.015), lending credibility to our results.

There are limitations to our study. After we launched this project, other SNPs were identified in obesity or T2D GWAS, including *MCR4* [10], *TCF7L2* [3, 4] and *EXT2* [7], but were not included in our study. In addition, population stratification can bias genetic association studies and produce spurious findings [18], and we only crudely accounted for potential population stratification by adjusting for self-reported race. However, given our null findings, population stratification does not appear to be an issue in our sample. Our study sample represents the general population in Kentucky, which is predominantly Caucasian. The small number of participants other than Caucasians (< 6%) limits our ability to estimate effects in other races.

Furthermore, we did not adjust for multiple testing in this study, but instead, examined these SNPs as novel candidate variants and tested *a priori* hypothesis of the existence of an association with colon cancer. Further adjustment for multiple testing would not alter our conclusion of no association with any of these SNPs and colon cancer.

Overall, we do not find evidence of shared genetic risk factors for colon cancer with obesity or T2D. However, the strong epidemiological and biological evidence for common underlying mechanisms influencing T2D and cancer warrants further investigation.
